# Application of Artificial Intelligence Techniques for the Estimation of Basal Insulin in Patients with Type I Diabetes

**DOI:** 10.1155/2020/7326073

**Published:** 2020-11-03

**Authors:** Guillermo Edinson Guzman Gómez, Luis Eduardo Burbano Agredo, Veline Martínez, Oscar Fernando Bedoya Leiva

**Affiliations:** ^1^Fundación Valle del Lili, Departamento de Endocrinología, Cali, Colombia; ^2^Universidad Icesi, Facultad de Ciencias de la Salud, Cali, Colombia; ^3^Universidad del Valle, Cali, Colombia; ^4^Universidad del Valle, School of Computer Science and Systems Engineering, Cali, Colombia

## Abstract

Artificial intelligence techniques have been positioned in the resolution of problems in various areas of healthcare. Clinical decision support systems developed from this technology have optimized the healthcare of patients with chronic diseases through mobile applications. In this study, several models based on this methodology have been developed to calculate the basal insulin dose in patients with type I diabetes using subcutaneous insulin infusion pumps. *Methods*. A pilot experimental study was performed with data from 56 patients with type 1 diabetes who used insulin infusion pumps and underwent continuous glucose monitoring. Several models based on artificial intelligence techniques were developed to analyze glycemic patterns based on continuous glucose monitoring and clinical variables in order to estimate the basal insulin dose. We used neural networks (NNs), Bayesian networks (BNs), support vector machines (SVMs), and random forests (RF). We then evaluated the agreement between predicted and actual values using several statistical error measurements: mean absolute error (MAE), mean square error (MSE), root-mean-square error (RMSE), Pearson's correlation coefficient (*R*), and determination coefficient (*R*^2^). *Results*. Twenty-four different models were obtained, one for each hour of the day, with each chosen technique. Correlation coefficients obtained with RF, SVMs, NNs, and BNs were 0.9999, 0.9921, 0.0303, and 0.7754, respectively. The error increased between 06:00 and 07:00 and between 13:00 and 17:00. *Conclusions*. The performance of the RF technique was excellent and got very close to the actual values. Intelligence techniques could be used to predict basal insulin dose. However, it is necessary to explore the validity of the results and select the target population. Models that allow for more accurate levels of prediction should be further explored.

## 1. Introduction

Diabetes mellitus (DM) is a chronic disease whose prevalence is on the rise and has been called the epidemic of the 21st century. DM has a great implication on morbidity and mortality. This disease represents high costs derived from the care of patients and the complications resulting from poor control of the disease [1]. Type 1 DM (T1DM) is characterized by low insulin production by the pancreas and the treatment is based on an exogenous replacement. Insufficient insulin in the body can lead to serious complications, such as ketoacidosis and hyperosmolar status that can lead to a diabetic coma and death [2, 3].

Many factors can affect blood glucose levels such as exercise, viral diseases, stress, and hormonal effects, which are not related to periods of food intake or insulin bolus but might be present during the time that basal insulin must cover. These events directly impact blood glucose levels in T1DM patients, hence the importance of establishing a correct basal insulin dose from the beginning of the treatment while facilitating the adjustment of the dose corresponding to boluses and sensitivity. Currently, medical guides propose insulin schedules based on the weight and age of the patient without taking into account other factors, such as those mentioned above [4]. Furthermore, very often, insulin adjustments end up being established following simple rules based on empiricism and the experience of the treating physician and are implemented late due to the difficulty the patient faces accessing specialized consultations.

In emerging countries like Colombia, access to healthcare services is limited. In this regard, clinical decision support systems based on artificial intelligence techniques could be useful as a strategy to close the care gaps [5–9]. Currently, different models have been proposed to estimate the basal insulin dose using artificial intelligence algorithms and techniques [10–18]. These models have shown good results in improving the time in the range of glycemic control [10–13]. Consequently, in this study, we aimed to evaluate different artificial intelligence models calculating basal insulin doses and estimates which could be better.

## 2. Material and Methods

A retrospective cohort study was conducted analyzing data from adult patients over 18 years with a diagnosis of T1DM using Medtronic 640G and Paradigm Veo insulin infusion pumps coupled to continuous glucose monitoring using Enlite sensor and with an acceptable glycemic control, defined by a range of glycated hemoglobin (HbA1c) between 6% and 8% and sensor use more than 80% were included. Comorbidities were not considered to excluded patients. All were treated at the Valle del Lili Foundation, located in Cali, Colombia.

Variables such as age, sex, weight, height, body mass index, glycated hemoglobin, basal insulin dose, upper and lower bounds of the standard deviation of the insulin dose, total insulin dose, percentage of basal insulin use and the daily dose of basal insulin, interstitial glucose, and upper and lower bounds of the standard deviation of interstitial glucose measurements were included. Data were obtained from the medical records collected using Medtronic Carelink Pro Version 4.0c software with a two-week data downloaded and supplemented with the clinical histories of the selected patients. For data analysis, we excluded days where high variability in blood glucose levels was observed (presence of acute disease, use of temporary basal conditions, hyperglycemia due to poor carbohydrate count, etc.).

### 2.1. Models for Estimating Basal Insulin Dose

Four artificial intelligence techniques were explored to estimate the basal insulin dose for each of the 24 hours in a day: neural networks (NNs), Bayesian networks (BNs), random forests (RF), and support vector machines (SVMs). The techniques were selected based on their ease of implementation in the R programming language. The artificial intelligence techniques selected are known as machine learning strategies. They create an insulin dose prediction model based on information from patients who are part of a training set. The model is capable of predicting insulin dose values for patients to different test set from the training set and therefore, could be generalized. The prediction model varies according to the machine learning technique. Machine learning strategies do not use a simple mathematical formula to predict a patient's dose. Instead, they train a model presenting values that correspond to insulin doses of patients in a training set. In this way, the model is able to learn the relationship between variables that represent each patient and insulin dose in order to predict the dose in patients from a test set different from the training set. For training, testing, and cross-validation of the models, we used the information of 56 patients, from whom a set of 640 records was obtained corresponding to the glycemic data for 15 days and for each of the 24 hours in a day.

### 2.2. Model Based on Neural Networks

Artificial neural networks are computational models analogous to biological neural networks present in the nervous system of living beings. Neural networks are made up of nodes or units connected through directed connections. A connection from unit “*j*” to unit “*I*” serves to propagate activation “*a*” from “*j*” to “*I*”. Furthermore, each connection has an associated numerical weight “*W*_*j*,*i*_*,”* which determines the strength and sign of the connection. Each unit “*i*” first calculates a weighted sum of its inputs:

(1) $$\begin{array}{c} in_{i}=\sum_{j=0}^{n}W_{j,i}a_{j}. \end{array}$$

Then an activation function *g* is applied to this sum to produce the output:

(2) $$\begin{array}{c} a_{i}=g\left(in_{i}\right)=g\left(\sum_{j=0}^{n}W_{j,i}a_{j}\right). \end{array}$$

**Figure 1:**
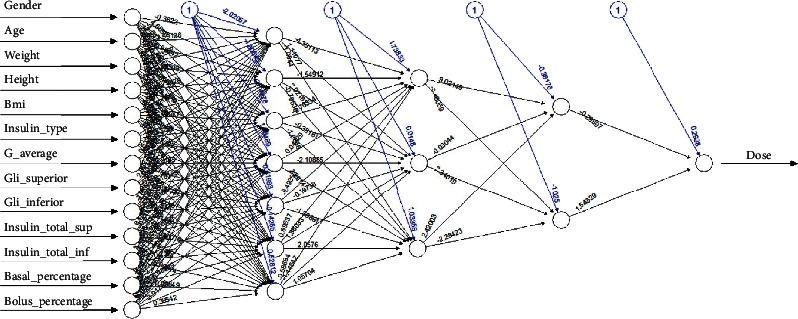
Structure of the neural network model 13-7-3-2.

The model based on the NNs technique was obtained using the neural net library in R. The network has 13 input neurons that correspond to the 13 aforementioned variables [11, 12]. Different topologies with different configurations were used and a 13-7-3-2-1 network topology was selected, that is, 13 input neurons, corresponding to the variables mentioned above, and 7, 3, and 2 neurons in hidden layers. Finally, in the output layer, only one neuron called Dose is obtained. The heuristic used was (*n* + *m*)/2, where *n* corresponds to the number of inputs and *m* to the number of output neurons. For example, for the first hidden layer, we have (13 + 1)/2. For this reason, 7 is the number of neurons in the first hidden layer. For the following layers, empirical adjustments determined from the results were performed. The best results were obtained by implementing the stochastic average gradient (SAG) algorithm, which is based on the Grprop algorithm and also derives from the electropropagation algorithm, defining the learning rate with the smallest absolute derivative without backward movement. The topology used is presented in Figure 1.

### 2.3. Model Based on Bayesian Networks

A Bayesian network is a directed acyclic graph made up of nodes and edges, where the nodes represent the random variables and the edges represent the probabilistic dependence that exists between them. The Bayesian network is capable of adjusting the probabilities between nodes in order to determine the output value of a variable to predict an insulin dose. The dependent variables correspond to variables used to represent patients. The Bayesian network as a statistical model facilitates the description and visualization of the probabilistic interrelationships between a set of variables of interest.

**Figure 2:**
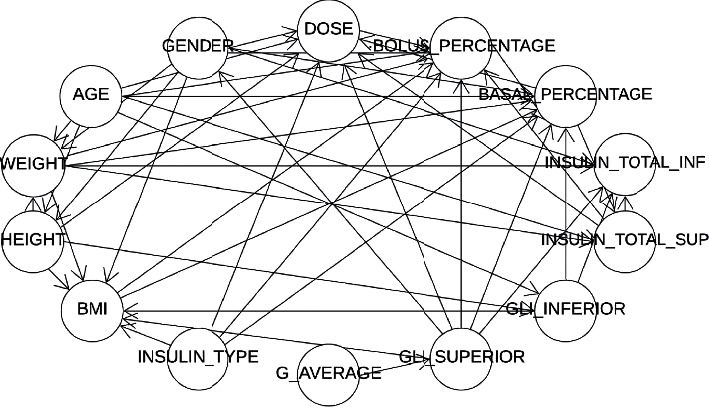
Dependencies in the Bayesian network model.

The BN obtained has a number of nodes corresponding to the variables that determine the insulin dose to be calculated. The bnlearn library was used within R. The dependencies between the nodes were automatically determined by the library. For example, in Figure 2, each circle is a node of the network and corresponds to one of the variables used by the prediction model, including the one to be calculated (dose). Each arrow directed to this node represents its direct dependence on each of the other attributes. However, this does not mean that there are no dependencies with the other characteristics, but rather that there is a stronger relationship of these characteristics with DOSE (Figure 2).

### 2.4. Model Based on Support Vector Machines

The Support Vector Machines technique builds an optimal hyperplane in the form of a decision surface so the margin of separation between the different classes in the data is as wide as possible. Based on labeled data represented as points or vectors in a dimensional space, the algorithm outputs have a hyperplane that can classify new data instances. Support vectors refer to a small subset of the training observations that are used as support for the optimal location of the decision surface. To build the separation between the hyperplanes, SVMs rely on a function called kernel and we used polynomial type:

(3) $$\begin{array}{c} K\left(x_{1},x_{2}\right)={\left(x_{1}^{T}x_{2}+1\right)}^{\rho }, \end{array}$$

where *ρ* is the order of the polynomial.

The vector machine model was obtained using the e1071 library in R and the polynomial function was used as the definitive kernel because it is the function that allows the separation of data to have a more accurate prediction. Similarly, tests were performed with the linear and sigmoid kernel functions. The polynomial kernel was adjusted with a constraint violation cost greater than 1, an epsilon insensitive loss function greater than 0.1, and a termination criterion tolerance greater than 0.001. The obtained SVMs model has a total of 13 classes which correspond to the variables used to make the prediction and 163 support vectors.

### 2.5. Model Based on Random Forests

The Random Forest technique combines multiple decision trees to predict a value more accurately than if you were to predict a single tree. Each tree predicts the value of the insulin dose using all variables that represent each patient. Finally, the technique is capable of average values predicted by all trees. The error generalization in this technique converges to a limit when the number of trees in the forest is large. Each tree makes a prediction independently and the technique averages those predicted values to have a single output value.

In the training stage, the algorithm tries to optimize the parameters of the split functions from the training samples:

(4) $$\begin{array}{c} \theta_{k}^{*}=\arg\max_{\theta j\varepsilon \tau j}I_{j}. \end{array}$$

For this, the following information gain function is used:

(5) $$\begin{array}{c} I_{j}=H\left(j\right)-\sum_{i\varepsilon 1,2}\frac{\left|S_{j}^{i}\right|}{\left|S_{j}\right|}H\left(S_{j}^{i}\right), \end{array}$$

where *S* represents the set of samples in the node to be divided, and *S*^*i*^ are the two sets that are created from the decision. The function measures the entropy of the set and depends on the type of problem that is being addressed.

**Figure 3:**
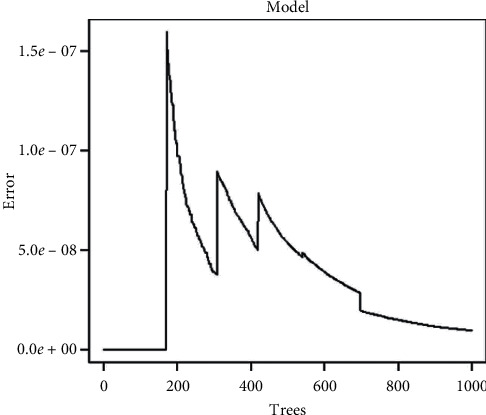
Representation of the prediction error using Random Forest.

For the Random Forest model, we used the Random Forest library in R, which implements Breiman's random forests algorithm. A sample with the 13 input variables starts a tour through various binary decision trees, which can come together in multiple solutions. In the end, these solutions are averaged to estimate the insulin dose. The number of decision trees generated for this purpose was adjusted to 1000. This value was chosen as the maximum number of trees to generate, which is determined from the moment the error stabilizes, as shown in Figure 3.

## 3. Result Analysis

We evaluated the concordance of the four artificial intelligence techniques in the calculation of the basal insulin dose in relation to the dose established by the physician defined in the data download of the insulin pump. Metrics used to measure accuracy were as follows: mean absolute error (MAE), mean squared error (MSE), root-mean-square error (RMSE), Pearson's correlation coefficient (*R*), and coefficient of determination (*R*^2^).

## 4. Results

Only 58 out of 480 patients had a regular follow-up at the institution from 2012 to 2018 and an acceptable glycemic control, i.e., a range of HbA1c between 6% and 8%. About 1456 records were stored for each patient. This data, a total of 81,536 records, were used to train the models (see Table 1).

**Figure 4:**
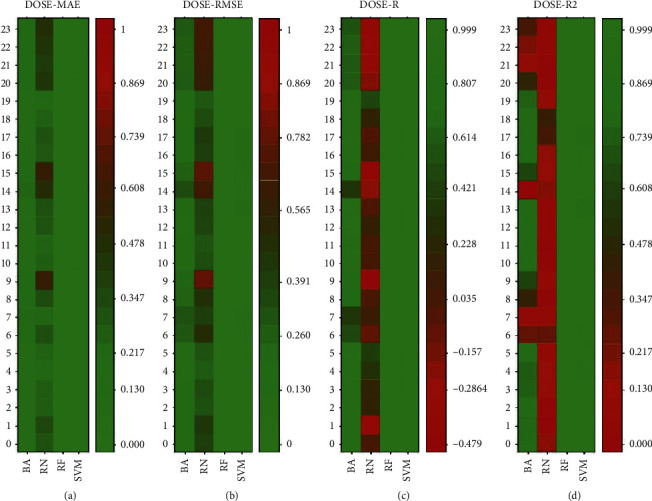
The metrics used to measure the accuracy of the four artificial intelligence techniques for the 24 subsets of data for dose calculation (a). Mean absolute error (b). Root-mean-square error (c). Pearson's correlation coefficient (d). Coefficient of determination.

The metrics used to measure the accuracy of the four techniques employed are shown using a color scale in Figure 4. The mean absolute error (MAE) of the four techniques (BNs, NNs, RF, and SVMs) is plotted on the *X*-axis and the 24 experiments (designed for each model as indicated in 2.4) are plotted on the *Y*-axis.

As observed in Figure 4(a), the BN technique reaches mean absolute errors (MAE) close to 1 which are represented in red. It indicates that the BN technique is not able to predict basal insulin doses specifically in certain hours such as 9 am, 2 pm, 3 pm, and between 8 pm and 11 pm. On the other hand, the RF and SVM techniques obtained mean absolute errors close to 0 which are presented in light green. Figure 4(a) allows seeing that RF and SVM techniques are more suitable for predicting basal insulin doses than the Bayesian networks and Neural networks.  Figure 4(b) shows the color scale for the root-mean-square error (RMSE). In this case, similar results are obtained. However, the SVM technique shows some values between 2 am and 7 am, and between 1 pm and 5 pm that have a dark green color which indicates that the RMSE values are not very close to 0. It should be observed that light green color is obtained in all hours for the RF technique. It means that Random Forest is better than BN, RN, and SVMs for predicting basal insulin doses.  Figure 4(c) shows the color scale for the Pearson's correlation coefficient (*R*). In this figure, the green color represents a correlation close to 1.0. It occurs when the predicted and the actual values are highly correlated. A Person correlation coefficient of +1 is called a total positive linear correlation and it shows that a technique is suitable in a specific prediction problem. As observed, all values are light green in RF and SVM which indicates an excellent correlation between the predicted and actual values. On the other hand, BN and RN have some negative correlation values that are presented in red. Finally, Figure 4(d) shows the color scale for the coefficient of determination (*R*^2^). The coefficient of determination measures how well a technique approximates the actual insulin doses. An *R*^2^ of 1 indicates that a technique perfectly fits the data. As observed, Random Forest presents all *R*^2^ values in light green. It means that RF is the best technique for predicting insulin doses. SVM has just a few hours in a darker green color. The BN and RN techniques have a poor coefficient of determination.

In the Table 2, we present a summary of the metrics used to measure the accuracy of the five experiments using the four techniques. The results obtained in the statistical error measurements show that the BNs and NNs techniques do not estimate the dose properly, while RF and SVMs had an excellent behavior getting very close to actual values with a MAE of 0.00006574310642 for RF and 0.01282676086 for SVMs. It was also evidenced that the error increases in certain periods of time, from 6 am to 7 am and between 13 pm to 17 pm. Pearson's correlation coefficient (*R*) with RF, SVMs, NNs, and BNs, were 0.9999, 0.9921, 0.0303, and 0.7754, respectively. As explained before, the values obtained by RF and SVM are considered a total positive linear correlation. It shows that RF and SVM are excellent choices when predicting insulin doses. The coefficient of determination (*R*^2^) of RF, SVMs, NNs, and BNs, was 0.9999, 0.9843, 0.0671, and 0.6348, respectively. The R^2^ value obtained by the RF technique is very close to 1.0 and it means that Random Forest is highly accurate to approximate the actual insulin doses.

**Figure 5:**
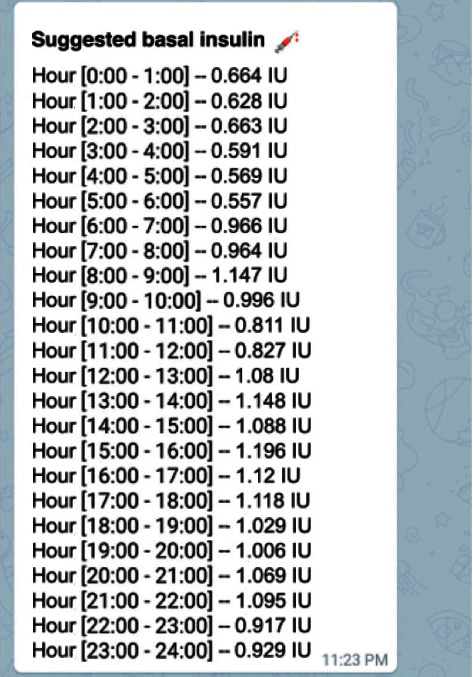
Example of basal dose insulin created by models.

In total, 96 artificial intelligence models were designed. It means 24 different models for each technique used, considering that the pump model used in the study allows a maximum of 24 basal insulin doses to be configured. The 24 models provide a basal insulin dose for each hour. Although these doses are not necessarily different, according to the tests carried out, there may be several doses per hour that can constitute a single dose range.  Figure 5 shows an estimate of 24 insulin doses calculated for 24 hours a day in a patient and how the dose estimated in some range of times were similar, for example, the estimated doses between 18:00-19:00 =  1,029 IU/hr. and 19:00–20:00=  1,006 IU/hr.

## 5. Discussion

DM is a highly prevalent condition worldwide with many nuances and to control it, a multidisciplinary approach with patient participation is required. Therefore, it is important to create strategies which allow the empowerment of patients to achieve glucose level goals avoiding hyperglycemia or hypoglycemia and their impact on morbidity and mortality. To this end, an adequate calculation of the basal insulin dose is an important strategy. Clinical decision support systems have been emerging as alternative methods in the care of patients with chronic diseases, such as diabetes. However, a comprehensive validation process is required [19, 20] for their application in patients and to achieve the proposed objectives.

Currently, different models have been proposed to predict the basal insulin dose using algorithms and artificial intelligence techniques [10–18]. Torrent-Fontbona proposes a system that calculates boluses with case-based reasoning and that in combination with Kalman filtering for baseline estimation reaches a time in the range of 83.87 + 1.35 [10, 11]. Meanwhile, Cappon et al. [12] use a NN to calculate insulin bolus with a reduction in the blood glucose risk index of 0.37, 0.23, and 0.20 versus the standard methods Scheiner, Pettus, and Edelman, respectively. In the study carried out by Nimri et al. [13] with 25 patients using fuzzy logic, most patients had numerical differences between −0.5 and 6 units with respect to the total daily dose of insulin prescribed by the medical specialist. However, there were cases where the difference was greater. So far, the results have been good, for example, in one of these cases, it was possible to go from an average glycemia of 259.43 ± 23.42 mg/dL to 156.73 ± 14.41 mg/dL.

In our case, of all the models obtained, The RF technique was found to be the most suitable for estimating the basal insulin dose and the dose estimated in some range of times was similar this according to the configuration that allows the insulin pump model 640G. Further, the error was greater in certain periods of time, between 6 am and 7 am and between 1 pm and 5 pm. This may be because they are the periods with the highest glycemic variability (GV), an integral component of glucose homeostasis [21]. Diet plays an important role in the variation of glycemia [22–24]. This concurs with the periods when calculating the basal insulin dose was less successful and coincided with breakfast in the morning and lunch in the afternoon. It will be important to join strategies in the future to optimize the calculation of ingested carbohydrates. Additionally, there might be other factors related to GV that should be taken into account to improve the model, such as stress, exercise, comorbidities, and the use of other medications.

Despite the fact that the number of patients seems small, records used for model training are much larger and more representative of the patients. A total of 1456 records were stored for each patient. Thus, 81,536 records were used to train models. This training stage allows the models to have the ability to generalize and be applied for basal insulin dose prediction to patients who did not participate in the training while maintaining high accuracy. This means that the model emulates medical practice in patients with the same characteristics as those described in the manuscript. The main limitation of our study is that data were generated from a population with acceptable glycemic control and thus, we do not know how these models will behave in other populations with different age ranges, higher HbA1c levels, or a high GV. Taking this into account, the RF technique could be a useful tool for clinical decision support systems on basal insulin dose for insulin pump users in our population. Another limitation was the dose prediction between 06:00–07:00 and 13:00–17:00. It could be explained by carbohydrate intake and bolus insulin. Also, physical activity and many regulatory hormonal secretions changes could affect glucose levels and basal insulin. It was our first approach to calculate basal insulin through artificial intelligence models. However, we need to work on the construction of models that predict basal and bolus insulin to improve prediction and get a better level of glycemic control. Anyway, the current error could be acceptable for basal insulin dose prediction but validation studies in other populations are necessary.

## Figures and Tables

**Table 1:** Demographic data.

Characteristics	Average value	Standard deviation
Age (years)	39.81	11.56
Women (%)	77.4	NA
Weight (kg)	65.81	8.45
Height (cm)	162.96	8.73
BMI (kg/m^2^)	24.77	2.61
Total insulin dose (UI)	37.78	15.5
Basal insulin (IU/hr)	0.908	0.43
%basal insulin	51.23	10.12
Interstitial glucose (mg/dL)	138.19	12.99
HbA1c (%)	7.1	0.66

NA = not applicate.

**Table 2:** Metrics used to measure the accuracy of experiments for dose calculation.

Technique	MAE	MSE	RMSE	*R*	*R* ^2^
BNs	0.1199083333	0.03133645935	0.1770210704	0.7754166667	0.634873650
NNs	0.3369750000	0.20192390070	0.4493594338	−0.0303916666	0.067199665
RF	0.0000657431064	0.00000037425226	0.000611761611	0.9999964346	0.999992869
SVM	0.01282676086	0.001109456742	0.03330850856	0.9921356180	0.984373906

MAE: mean absolute error. MSE: mean squared error. RMSE: root-mean-square error. *R*: Pearson's correlation coefficient. *R*^2^: coefficient of determination. BNs: Bayesian networks. NNs: neural networks. RF: Random Forest. SVMs: support vector machines.

## Data Availability

Supporting information is available from the authors upon request by e-mail to guillermoeguzman@gmail.com.
